# Ethyl Cellulose-Core, OSA Starch-Shell Electrosprayed Microcapsules Enhance the Oxidative Stability of Loaded Fish Oil

**DOI:** 10.3390/nano14060510

**Published:** 2024-03-12

**Authors:** Elnaz Z. Fallahasghari, Peter Reimer Stubbe, Ioannis S. Chronakis, Ana C. Mendes

**Affiliations:** DTU—Food, Research Group for Food Production Engineering, Laboratory of Nano-BioScience, Technical University of Denmark, Henrik Dams Allé, B202, 2800 Kongens Lyngby, Denmark; zeyfal@food.dtu.dk (E.Z.F.); prst@food.dtu.dk (P.R.S.)

**Keywords:** ethyl cellulose, OSA–modified starch, fish oil, micro-encapsulation, coaxial electrospray, oxidative stability

## Abstract

The encapsulation and the oxidative stability of cod liver fish oil (CLO) within coaxial electrosprayed (ethyl cellulose/CLO) core–(octenyl succinic anhydride, OSA-modified starch) shell, and monoaxial electrosprayed ethyl cellulose/CLO microcapsules were investigated. Core–shell (H-ECLO) and monoaxial (ECLO) electrosprayed microcapsules with an average diameter of 2.8 ± 1.8 µm, and 2.2 ± 1.4 µm, respectively, were produced. Confocal microscopy confirmed not only the core–shell structure of the H-ECLO microcapsules, but also the location of the CLO in the core. However, for the ECLO microcapsules, the CLO was distributed on the microcapsules’ surface, as also confirmed by Raman spectroscopy. Atomic force microscopy showed that the average surface adhesion of the H-ECLO microcapsules was significantly lower (5.41 ± 0.31 nN) than ECLO microcapsules (18.18 ± 1.07 nN), while the H-ECLO microcapsules showed a remarkably higher Young’s modulus (33.84 ± 4.36 MPa) than the ECLO microcapsules (6.64 ± 0.84 MPa). Differential scanning calorimetry results confirmed that the H-ECLO microcapsules enhanced the oxidative stability of encapsulated CLO by about 15 times, in comparison to non-encapsulated oil, mainly by preventing the presence of the fish oil at the surface of the microcapsules, while ECLO microcapsules enhanced the oxidative stability of CLO about 2.9 times due to the hydrophobic interactions of the oil and ethyl cellulose. Furthermore, the finite element method was also used to evaluate the electric field strength distribution, which was substantially higher in the vicinity of the collector and lower in the proximity of the nozzle when the coaxial electrospray process was employed in comparison to the monoaxial process.

## 1. Introduction

The awareness of both researchers and consumers about the health benefits of omega-3 polyunsaturated fatty acids (n–3 PUFAs) has been increasing in the last few years [1,2]. PUFAs have anti-inflammatory properties and can reduce depressive symptoms and neurodegenerative diseases, including Alzheimer’s and Parkinson’s [3]. PUFAs are also beneficial to acute pancreatitis patients [4], the development of the brain, the central nervous system, and the visual response in the brain system [5,6,7]. Moreover, omega-3 fatty acids balance lipid metabolism and reduce the risk of cardiovascular disease [8,9] and some types of cancer [9,10] and arthritis [11,12]. Omega-3 long-chain fatty acids, such as eicosatetraenoic (EPA; 20:5n–3) and docosahexaenoic (DHA; 22:6n–3), are essential in fetal development and precursors of several metabolites [13].

However, a significant majority of the world’s population (81.1%) consumes a lower daily intake than the recommended value (250 mg) [14]. The main sources of EPA and DHA are marine animals and plants [13]. The human body can synthesize an extremely limited number (2–10%) of alpha-linolenic acid (ALA) to EPA and DHA via elongase and desaturase enzymes. Therefore, having an appropriate intake of EPA and DHA is essential [13] for a healthier human metabolism. Fish oil is one of the most conspicuous natural sources of PUFAs including EPA and DHA [15,16]. The EPA and DHA content can reach 35% depending on the fish species [14]. Thus, food fortification with fish oil is valuable for increasing the consumption of omega-3 PUFAs.

The high amount of unsaturated fatty acids in fish oil make it susceptible to oxidation, resulting in loss of functional and nutritional properties. During the oxidation of fish oil, the development of oxidation products such as peroxides, aldehydes, ketones, and their volatile compounds takes place. Those usually impact the flavor and the health-related properties of the product [17,18]. 

To defeat this problem, the encapsulation of fish oil is predominantly used in the food and pharmaceutical industries. This enables them to manufacture fish oil with higher oxidative stability and incorporate it into various products [19]. Encapsulation can protect fish oil from light, heat, water, pH changes, and enzymes to inhibit undesirable reactions such as oxidation [20,21]. In addition, encapsulation can be beneficial in masking unwanted taste or odor of fish oil [20]. Therefore, the food industry is constantly seeking encapsulation strategies for fish oil that allow its incorporation into food products [8,9].

Electrohydrodynamic (EHD) stands out as a promising method for the encapsulation of fish oil [22], as the encapsulation process can be carried out at room temperature and therefore can prevent the fish oil from oxidation [23,24]. Furthermore, it is known for having relatively high encapsulation efficiency [20,25]. Encapsulation of fish oil with monoaxial electrospray processing has been achieved using different food-grade polysaccharides (e.g., gelatin [26], glucose [20], dextran, pullulan, and glucose syrup [20,27,28] as well as proteins such as zein [18] and kafirin [29]) and using various solvents (water, alcohol, and acetic acid) [19,30]. Alternatively, coaxial electrospraying allows for the production of core–shell capsules, where a protective layer of shell compounds surrounds the core-encapsulated compound(s). Contrary to monoaxial electrospray capsules, where the fish oil can be exposed at the surface of the microcapsule, the core–shell capsules allow a negligible amount of surface oil, if any. Consequently, the encapsulation of the fish oil in the core–shell capsules enhances its oxidative stability [22,31].

Despite the potential of coaxial electrospraying, to the best of our knowledge, only a few studies investigated core–shell capsules for the encapsulation of fish oil. Rahmani-Manglano et al. investigated the oxidative stability of encapsulated fish oil using monoaxial and coaxial processing using low-molecular-weight carbohydrates (glucose syrup or maltodextrin). Fish oil encapsulated within coaxial electrosprayed (ES) microcapsules showed lower oxidation than fish oil encapsulated within monoaxially electrosprayed capsules [32]. Furthermore, electrosprayed core–shell microcapsules can enhance the oxidative stability of other sensitive lipophilic compounds [33]. 

Starch and cellulose are the most abundant and neutral plant-derived polysaccharides [20]. Among cellulose derivatives, ethyl cellulose has been used for the electrohydrodynamic encapsulation of bioactive compounds and probiotics [34,35,36]. Likewise, starch in native and modified forms has been used for the encapsulation of a range of sensitive food bioactive compounds [37]. Du et al. produced electrospun nanofibers of OSA–starch, pullulan (PUL), and hydroxypropyl–beta–cyclodextrin (HPβCD) and observed high antioxidant activity properties in encapsulated curcumin [38]. Biduski et al. used OSA–starch (Capsul^®^) for the encapsulation of rosemary essential oil emulsions by electrospraying using different concentrations of aqueous ethanol (20%, 30% and 40% *v*/*v*). The ethanol content of the emulsions affected the emulsion’s stability, as well as the morphology, loading capacity, and encapsulation efficiency of the electrosprayed microparticles. Fourier-transform infrared spectrometry suggested the interaction of essential oil with OSA–starch [39]. Electrospun fiber mats of OSA–starch (Hi–cap 100, purity Gum Ultra, purity Gum 2000) and pullulan (PUL) have been evaluated for the adsorption of the odor compounds of oyster peptides. X-ray diffractometry and Fourier transform infrared (FTIR) analysis confirmed that the PUL and OSA–starches interacted, changing the nanofiber mats from amorphous structures to weak V-type crystal structures. Moreover, XRD and FTIR confirmed that the electrospun PUL and OSA–starch nanofiber mats could adsorb volatile components of oyster peptides in a solid phase as well as showing a good adsorption effect for odor compounds [40]. In a recent study, cinnamaldehyde essential oil (CEO) was loaded into electrospun octenyl succinylated starch–pullulan nanofiber mats using six different OSA–starch variants with different molecular structures from potato and waxy maize. The CEO-loaded nanofiber mats exhibited antibacterial effects against *Staphylococcus aureus, Escherichia coli,* and *Aspergillus flavus* microorganisms [41].

Our previous study showed that electrosprayed core–shell microcapsules made of a shell of octenyl succinic anhydride (OSA) modified corn starch, maltose (Hi–Cap), and a core of ethyl cellulose with vitamin A palmitate (AP) enhanced the oxidative stability of the encapsulated AP [33]. The present study investigated the development of ethyl cellulose core—OSA-modified starch shell electrosprayed microcapsules for the encapsulation of cod liver fish oil. Monoaxial electrosprayed microcapsules of the core compounds (ethyl cellulose and oil) were also developed. The oxidative stability of CLO encapsulated within both monoaxial and coaxial electrosprayed microcapsules was evaluated using differential scanning calorimetry (DSC). The physicochemical properties of the microcapsules were also assessed using Raman and attenuated total reflectance–Fourier transform infrared (ATR–FTIR) spectroscopy and atomic force microscopy (AFM) methods.

## 2. Materials and Methods

### 2.1. Materials

Ethyl cellulose (48.0–49.5% *w*/*w*) ethoxyl basis (E), and Span^®^ 80 (Sorbitane monooleate), Nile blue A (Nile blue sulfate), Nile red (Nile blue A oxazone), and acetone were purchased from Sigma-Aldrich Chemicals Co. (St. Louis, MO, USA). Cod liver oil (CLO) was acquired from MP Biomedicals, LLC (Solon, OH, USA). Hi–Cap^®^ 100 (OSA-modified corn starch (E 1450) containing 50% maltose (H) was obtained from Ingredion (Westchester, NY, USA). n–Hexadecane, 99% pure, was obtained from ACROS Organic (Geel, Belgium). Ethanol absolute ≥99.9% was obtained from VWR Life Science BDH (Rosny-sous-Bois, France) and Milli–Q^®^ water (Elix Technology Inside, Merck (Darmstadt, Germany) was used for preparing the electrospray solutions. 

### 2.2. Preparation of Electrospray Solutions

To prepare the microcapsule’s shell solution, Hi–Cap^®^ 100 (90% *w*/*v*) was dissolved into milli–Q water and constantly stirred using a magnetic stirrer (IKA-WERKE, RCT Basic, GmbH & Co. KG, Staufen im Breisgau, Germany) at 600 rpm for 4 h at room temperature (20 °C). The solution was allowed to stand overnight without stirring to remove the air bubbles. The core solution for the coaxial electrosprayed microcapsules was prepared using ethyl cellulose (8% *w*/*v*) dissolved in ethanol overnight, using a magnetic stirrer at 600 rpm. Span 80 surfactant (20% *w*/*w* with respect to fish oil) was mixed with the oil (10% *w*/*w*), and this mixture was then added to the ethyl cellulose solution and stirred in the dark for 30 min at 600 rpm prior the electrospray processing. For the monoaxial electrosprayed microcapsules, the same ethyl cellulose solution with Span 80 and oil was utilized. 

### 2.3. Microcapsule Production by Electrospraying 

The electrospray process was set horizontally and carried out on the lab scale. Core and shell solutions were loaded into the 10 mL disposable syringes and mounted on the two syringe pumps (New Era Pump System Inc., Farmingdale, NY, USA), which were connected to a three-fluid spray nozzle (Part of BUCHI B–290 Mini Spray Dryer B–290, BÜCHI Labortechnik AG, Essen, Germany). The nozzle consisted of a needle with an inner diameter of 0.7 mm and outer diameter of 1.5 mm for the core solution surrounded by a nozzle tip with an inner diameter of 2 mm for the shell solution. The outer diameter of the nozzle was 25 mm. For the monoaxial electrospray process, the needle part of the three–fluid spray nozzle was used. The flow rates of shell and core solutions were set at 0.01 mL/min and 0.005 mL/min, respectively. A high-voltage electrostatic field supplied by a high-voltage power supply (Gamma High Voltage Research, Ormono Batch, FL, USA), fixed at 40 kV, was applied between the nozzle and a stainless steel collector plate (30 cm × 30 cm) (covered with aluminum foil) separated by 30 cm. The electrospraying process was carried out at room temperature, i.e., 20 °C, inside a chamber with a controlled relative humidity of 35% ± 5 with nitrogen gas.

### 2.4. Characterisation of the ES Microcapsules

#### 2.4.1. Morphology 

The morphology of the microcapsules was studied by scanning electron microscopy (SEM) using a Quanta FEG 200 Cyro ESEM (environmental scanning electron microscope) (FEI Company, Hillsboro, OR, USA) at an accelerating voltage of 3 kV, a working distance of about 10 mm, and with a secondary electron detector (Everhart–Thornley detector–ETD). Approximately 0.5 cm × 0.5 cm of a thin layer of dried electrosprayed capsules on the aluminum foil were attached to SEM specimen stubs using carbon adhesive discs (Agar Scientific Ltd., Essex, UK). An automatic sputter coater (Q 150T Quorum Technologies Ltd., Lewes, UK) was used for coating the capsules. The capsules were coated with gold for a sputtering time of 20 s and a sputtering current of 20 milliamperes (mA). The dispersion and the size of the capsules were investigated in detail by utilizing the computer program ImageJ 1.47t, (National Institute of Health, Bethesda, MD, USA). For each sample, one hundred capsule sizes were measured randomly, analyzed and plotted using OriginPro 2021 9.8.0.200 (OriginLab Corporation, Northampton, MA, USA) software. 

#### 2.4.2. Localization of Oil Distribution within the Microcapsules 

Localization of oil distribution in the monoaxial and at the core–shell microcapsules was investigated by confocal laser scanning microscopy (CLSM) using an Inverted Zeiss LSM–710 microscope (Carl Zeiss MicroImaging GmbH, Jena, Germany) equipped with a diode laser (405 nm), argon laser (458, 488 and 514 nm), two HeNe lasers (543 and 633 nm), three detectors, and one transmitted detector. Images were acquired using a 40×/0.75 objective lens (EC–plan) and 514 nm and 633 nm excitation wavelengths. The fluorescence intensities across the diameter of the core–shell microcapsules were obtained using Zeiss Zen 2009 software (Carl Zeiss MicroImaging GmbH, Jena, Germany). 

For the preparation of the stains, we used stock solutions of 0.01% *w*/*w* Nile blue dissolved in Milli–Q^®^ water using the method described by [42] and 0.025% wt. of Nile red in acetone according to [43], with a mixing time of 30 min. Both solutions were prepared in amber bottles at room temperature. Prior to the electrospraying process, 10 µL of Nile red stock solution was added into 5 mL of the core solution, and 40 µL of Nile blue stock solution was added into 10 mL of core solution, with a mixing time of 30 min in the dark. The electrospraying was carried out in dim light. 

#### 2.4.3. Mechanical Properties of the ES Microcapsules 

The mechanical properties of the microcapsules were evaluated by a Bruker JPK NanoWizard^®^ 4 XP BioScience atomic force microscope (AFM) (Berlin, Germany). A Bruker scanning probe (cantilever), RFESPG–75, was used in a resonant frequency range of 50–100 kHz, and a spring constant of around 1.7 N/m. The topographical images were acquired in air and at ambient temperature in quantitative imaging (QI) mode with a scanning area of 20 µm × 20 µm for each image. The stiffness (i.e., Young’s modulus) and adhesion of the surface of the microcapsules (around 10 different areas) of both the monoaxial and coaxial microcapsules were obtained using JPK data processing software 2023 (version 8.0.98, Bruker, Berlin, Germany). The Young’s modulus, which is the elastic property of the material, can be calculated considering a purely elastic model obeying Hooke’s law [44] by Equation (1), when the sample is uniformly compressed or extended:

σ = 𝐸𝜀(1)

where σ is the stress, *ε* is the relative deformation to the height, and *E* is the Young’s modulus. However, in the case of a nonuniform pressure distribution, as can be in sphere–plane contact, the Hertzian pressure needs to be considered [45]. Thus, for small deformations, only the end of the cantilever tip is in contact with the sample, and the material can be considered a sphere compressed into a flat plane. For this geometry, the deformation *δ* is defined as

(2) $$\delta ={\left(\frac{3}{2}\right)}^{2/3}{\left(\frac{F^{2}}{{E^{\prime }}^{2}R}\right)}^{1/3}$$

where *F* is the force applied, *R* is the radius of the cantilever tip end, and *E*′ is the equivalent Young’s modulus, defined by

(3) $$E^{\prime }=2{\left(\frac{1-\nu_{c}^{2}}{E_{c}}+\frac{1-\nu_{t}^{2}}{E_{t}}\right)}^{-1}$$

where *E_c_* and *E_t_* are the Young’s modulus for the capsules and the cantilever tip, respectively, and *ν_c_* and *ν_t_* are the Poisson number for the capsules and the cantilever tip, respectively.

#### 2.4.4. Attenuated Total Reflection–Fourier Transform Infrared (ATR–FTIR)

FTIR analysis of the electrosprayed microcapsules, the core and shell compounds, and the core solution was carried out utilizing a Nicolet iS50 FT–IR (Thermo Fisher Scientific, Waltham, MA, USA). To gain higher FTIR spectra resolution, the ethanol of the core dispersion was evaporated under a nitrogen flow in the darkness. Additionally, the FTIR spectra of the microcapsules were measured and compared with the spectra of the individual compounds. For the FTIR measurements, approximately 10 mg of the samples were placed on top of the diamond ATR to cover it properly. All the spectra were acquired within a wavenumber of 4000–600 cm^−1^ with a resolution of 4 cm^−1^. Each spectrum was collected at 32 scans in absorbance mode.

#### 2.4.5. Raman Spectroscopy

The Raman spectra of electrosprayed microcapsules, non-encapsulated CLO, and ethyl cellulose and Hi–cap were obtained by using a DXR3 Raman microscope (Thermo Fischer Scientific, Waltham, MA, USA) equipped with Omnic 9.12.928 software. All the spectra were obtained with a laser wavelength of 532 nm, a preview exposure of 5 s, laser power of 10 mW, 10× magnification and 50 pinhole size, and aperture within the range of 4000 to 400 cm^−1^. 

#### 2.4.6. Encapsulation Efficiency 

The encapsulation efficiency (EE) of the microcapsules was determined following the conventional assay with some modifications [46,47]. This method is based on the removal of non-encapsulated oil, located on the surface of the nano-macro particles (NMS), with a proper solvent. Electrosprayed capsules (25 mg) were submerged in hexadecane (5 mL) and gently shaken with an IKA MS 3 digital shaker (IKA^®^—Works Inc., Wilmington, NC, USA) at 100 rpm for 15 min. After filtering the mixture through quantitative grade 1 filter paper with 11 µm particle retention (Whatman, Buckinghamshire, UK), the absorbance of the liquid was measured at 260 nm using a NanoDrop^TM^ One /OneC Microvolume UV–Vis spectrophotometer (Thermo Fisher Scientific, MA, USA). The amount of oil on the surface of sprayed capsules was determined from the calibration curve (R2 = 0.98) resulting from UV absorbance measurement of various dilutions of CLO in hexadecane. The EE value was measured as

(4) $$EE\left(\%\right)=\frac{A-B}{A}*100$$

where *A* is the theoretical amount of CLO in the formulation and *B* is the amount of CLO which is collected in the solution. The measurements were carried out in triplicate. 

#### 2.4.7. Oxidative Stability by Differential Scanning Calorimetry 

The oxidative stability of encapsulated CLO was evaluated in an oxygen atmosphere by the DSC isothermal method, using the DSC 250 (TA instrument Ltd., New Castle, UK) oxidation induction time (OIT) method described by Fallahasghari et al. [33]. The DSC was calibrated with high-purity indium standard. The weight of the sample was calculated to retain the same amount of oil for both the monoaxial and the coaxial electrosprayed microcapsules. Each sample was placed in an aluminum pan with an open lid and placed in the DSC instrument tray. Samples were heated under the nitrogen flow (50 mL/min) to the desired isothermal temperature with a heating ramp of 5 °C/min. An empty aluminum open pan was used as a reference. The isothermal experiment was conducted in oxygen (purity 99.995%) with a flow rate of 50 mL/min for 90 min. For the quantification of the oxidation of non-encapsulated CLO and encapsulated CLO of both monoaxial and coaxial electrosprayed microcapsules, the areas under the isothermal DSC curves were calculated. A calibration curve for oxidation of non-encapsulated CLO (1–13 mg) was used for comparison with the encapsulated CLO. The thermal stability of the CLO microcapsules was also evaluated at isothermal temperatures of 120 °C, 140 °C, and 160 °C.

### 2.5. Numerical Simulation of Electric Field 

The electric field strength and electromagnetic potential distribution between the nozzle tip and collector in the monoaxial and coaxial electrospray process were calculated with a numerical simulation, the finite element method (FEM), using a FreeFEM (version 4.13, Paris, France) adapted from [48]. 

### 2.6. Statistical Analysis

The statistical data analysis of the samples was performed with Statgraphics Centurion 19.6.02 (Statgraphics Technologies, Inc., The Plains, VA, USA) software. Statistical significance was determined by analysis of Tukey’s HSD, a multiple range test, and an ANOVA. The statistical significance between the two samples was determined by a two-sample comparison (*t*-test) in Microsoft 365 MSO Excel software (version 2402). A *p*-value under 0.05 was the criterion of significant difference between mean values, indicated by one asterisk (*); a *p*-value of less than 0.01 is represented by two asterisks (**), and a *p*-value of less than 0.001 is indicated by three asterisks (***) on the graphs. 

## 3. Results and Discussion

### 3.1. Morphology of the Microcapsules 

**Figure 1:**
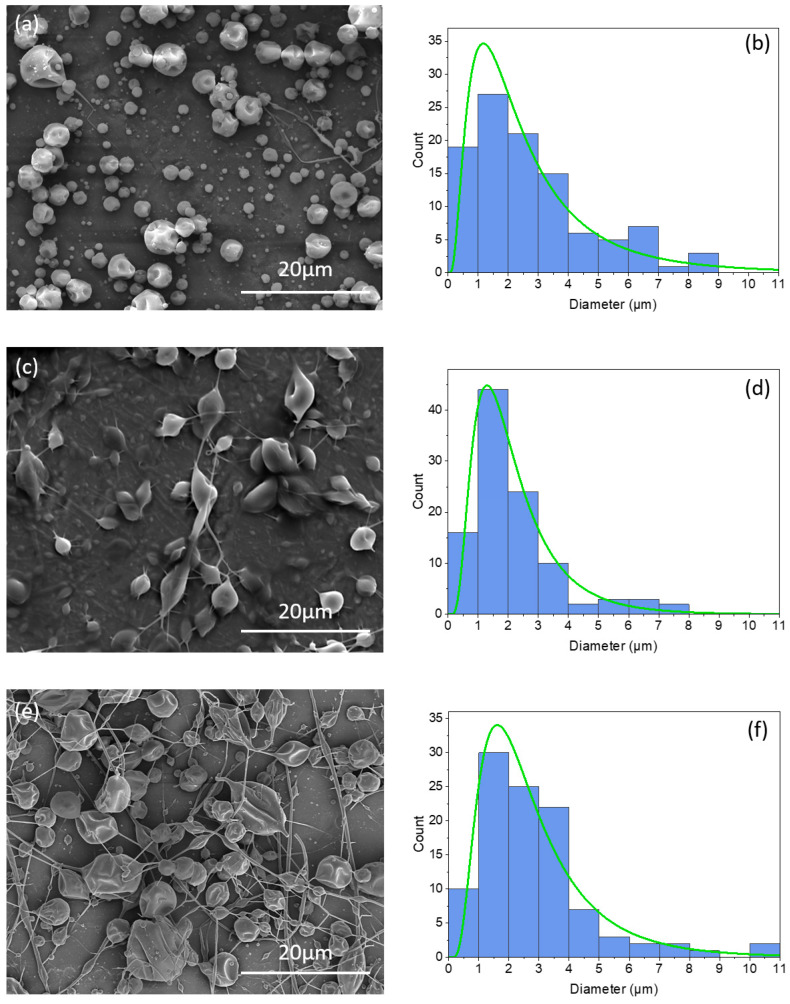
SEM images of (**a**) coaxial electrosprayed control microcapsules (H–E), (**c**) monoaxial electrosprayed microcapsules (ECLO), (**e**) coaxial electrosprayed microcapsules (H–ECLO), and (**b**,**d**,**f**) corresponding histograms with a diameter distribution of H–E, ECLO, and H–ECLO.

As shown in Figure 1a, the coaxial electrosprayed control microcapsules (without CLOin the core) (H–E) displayed a spherical shape morphology, while the monoaxial electrosprayed microcapsules loaded with CLO (ECLO) (Figure 1c) showed a semi-spherical shape with some fibers. However, the core–shell microcapsules loaded with CLO (H–ECLO) (Figure 1e) showed a semi-spherical shape connected with some fibers, with a slightly wrinkled, non-porous surface and relatively narrow capsule size distribution. Rahmani-Manglano et al. also observed fibers interconnecting the capsules for both monoaxial and coaxial electrosprayed fish-loaded microcapsules [32]. This morphology may be related to the viscosity and surface tension of the electrosprayed material solutions [49]. Moreover, the microcapsules showed a non-porous, fracture-free and smooth surface, which is preferred in order to eliminate oxygen diffusion, migration, and consequently, oil oxidation [50]. The microcapsules exhibited diameters ranging from 0.38 µm to 10.86 µm, with an average diameter of 2.7 ± 2.0 µm, 2.2 ± 1.4 µm and 2.8 ± 1.8 µm for control coaxial (H–E), monoaxial (ECLO), and coaxial microcapsules (H–ECLO), respectively (Figure 1b,d,f).

### 3.2. Localization of Oil by CLSM and EE of the Microcapsules

**Figure 2:**
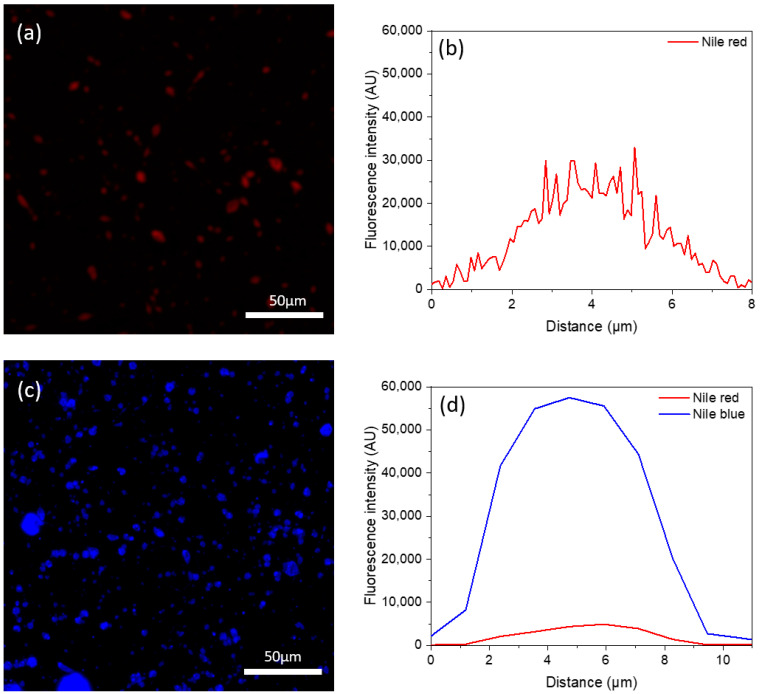
Confocal laser scanning microscopy (CLSM) image of (**a**) monoaxial electrosprayed microcapsules (ECLO), CLO stained with Nile red (red color), and (**b**) the respective fluorescence intensity profile across the diameter of the microcapsules. (**c**) CLSM image of coaxial electrosprayed microcapsules (H–ECLO), CLO stained with Nile red (red color), and shell compound stained with Nile blue (blue color). (**d**) The respective fluorescence intensity profile (for blue and red colors) across the diameter of the microcapsules.

The CLSM image of the monoaxial electrosprayed microcapsules (ECLO), (Figure 2a) showed that the stained CLO with red Nile (red color) dispersed on the surface of the microcapsules. The fluorescence intensity from the profile line across the capsule diameter (Figure 2b) confirms that the CLO is distributed not only at the surface but also in the entire area of the monoaxial microcapsules.

For the core–shell microcapsules (H–ECLO), the core of electrosprayed ethyl cellulose–CLO was stained with red Nile, while the shell of Hi–Cap starch was stained with Nile blue. Figure 2c shows a distinct shell of Hi–Cap (blue color) for the core–shell microcapsules, with no red color noticed on the surface. Furthermore, the fluorescence intensity of the coaxial microcapsules (Figure 2d) shows that the CLO was indeed encapsulated within the core. These results confirm the successful encapsulation of CLO within the core of the microcapsule.

The encapsulation efficiency of the electrosprayed microcapsules was 76.45 ± 1.10%, and 66.4 ± 3.60%, for the ECLO and H–ECLO, respectively. These results are similar to the EE of fish oil encapsulated within maltodextrin and glucose syrup electrosprayed microcapsules and whey protein concentrate hydrolysate as an emulsifier (EE < 72% for monoaxial and 53–59% for coaxial) [32].

### 3.3. 3D Surface Topography and Mechanical Properties of Microcapsules by AFM

**Figure 3:**
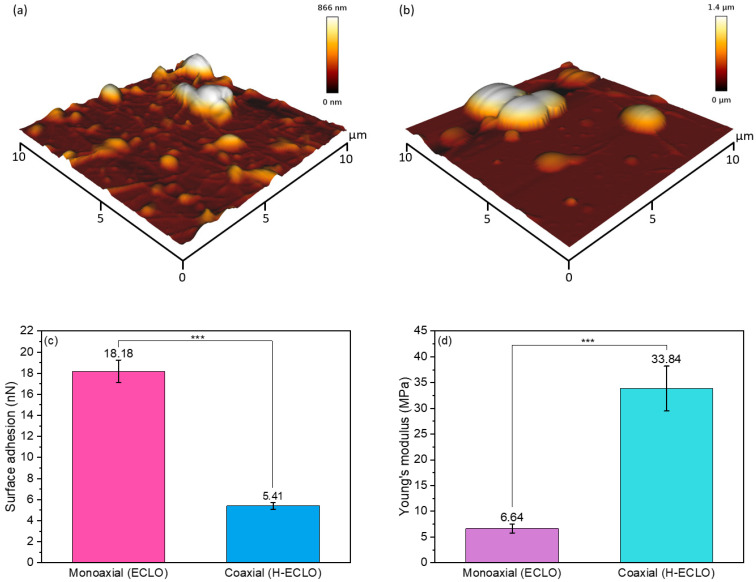
AFM data of the electrosprayed microcapsules. Representative 3D topographic images of the surface of the (**a**) monoaxial (ECLO) and (**b**) coaxial (H–ECLO) microcapsules. (**c**) surface adhesion, and (**d**) Young’s modulus. Three asterisks indicate significant differences with *p*-values of less than 0.001.

The 3D topographic images of the microcapsules’ surface, and their surface adhesion properties measured by AFM are shown in Figure 3a–c. The surface adhesion of the microcapsules was investigated by AFM (Figure 3c). The average surface adhesion of the coaxial electrosprayed capsules (H–ECLO) was 5.41 ± 0.31 nN, which was significantly lower than monoaxial electrosprayed capsules (ECLO), with a surface adhesion of 18.18 ± 1.07 nN. The lower surface adhesion for the coaxial microcapsules could be due to the hydrophilic shell of the OSA-modified starch, which had less affinity with the hydrophobic AFM tip. The surface adhesion results also suggest that the oil was well entrapped within the core–shell microcapsules, contrary to the monoaxial process. 

Furthermore, the H–ECLO microcapsules showed a remarkably higher Young’s modulus (33.84 ± 4.36 MPa) than the ECLO microcapsules (6.64 ± 0.84 MPa), as shown in Figure 3d. Probably this can be due to the elasticity of the OSA–starch shell of the microcapsules [51]. Besides, the lower elasticity determined for the ECLO microcapsules can be due to the presence of the surface oil. Campo et al. also observed a lower Young’s modulus for the corn starch films prepared using high safflower oil content [52]. 

### 3.4. FTIR Analysis

**Figure 4:**
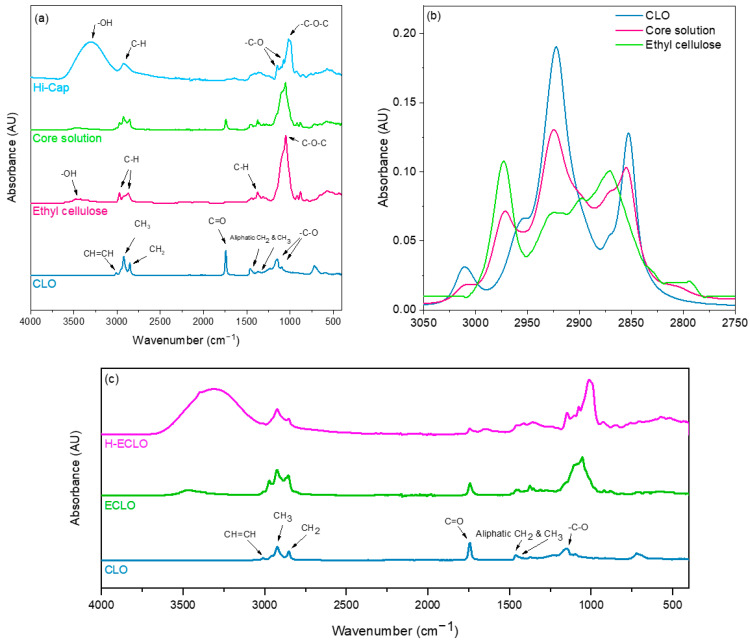
FTIR spectra of (**a**) cod liver oil (CLO), ethyl cellulose powder, and the core compounds of the microcapsules (after evaporation of ethanol); (**b**) the expanded region from 2750–3050 cm^−1^ and (**c**) FTIR spectra of monoaxial (ECLO) and coaxial (H–ECLO) electrosprayed microcapsules.

The FTIR spectrum of the CLO (Figure 4a) showed characteristic bands for alkane functional groups at wavenumbers of 2700–3000 cm^−1^, which are attributed to C–H stretching vibrations. The small peak around 3010 cm^−1^ indicates the C–H stretching of cis double-bond CH=CH groups (cis alkene HC=CH–) [47,53]. The intensive peak at 2923 and 2852 cm^−1^ is assigned to –C–H (CH_2_ and CH_3_ groups, symmetrical stretching). The peak at 1743 cm^−1^ corresponds to C=O carboxyl groups (triglycerides, ester stretching) [19,54]. The small peak at 1459 cm^−1^ contributed to the bending vibration of aliphatic –CH_2_– groups, while the peaks at 1376 cm^−1^ contributed to the bending vibration of aliphatic CH_3_ groups [47]. The bands at 1147 cm^−1^ and 1098 cm^−1^ correspond to carbonyl group –C–O stretching vibrations [55]. 

The ethyl cellulose spectrum showed main peaks at 3474 cm^−1^ due to the –OH groups of the polysaccharide backbone [56]. The asymmetric peaks of CH stretching can be seen in the peak regions of 2870–2970 cm^−1^. Other relevant vibration peaks around 1052 and 1375 cm^−1^ were mainly attributed to C–O–C stretching and C–H bending, respectively [57].

The FTIR spectrum of ethyl cellulose with CLO (core dispersion after evaporation of the solvent) showed main bands at 3474, 1054 cm^−1^ from the ethyl cellulose and a CLO band at 1743 cm^−1^ from C=O. However, the core dispersion spectra displayed shifts of bands within the region of 2850 to 3100 cm^−1^, which corresponds to CH stretching (Figure 4b), thus suggesting hydrophobic interactions between CLO and ethyl cellulose. In addition, the C–O–C stretch absorption band seen in the ethyl cellulose spectrum slightly changed in the core solution, which might be due to changes in the bond strength related to the use of ethanol as a solvent. 

The Hi–Cap spectra showed a broad peak at around 3324 cm^−1^, indicating the presence of hydroxyl group (–OH). Absorption at 2925 cm^−1^ is related to the C–H vibration of the glucose unit [58]. The peak at 1012 cm^−1^ corresponds to the C–O stretching of the C–O–C of the polysaccharide. The peaks at 1076 and 1148 cm^−1^ are attributed to anhydroglucose ring C–O stretching [59]. 

The FTIR spectra of the monoaxial microcapsules, ECLO (Figure 4c) showed similar spectra to the spectra of the core dispersion (Figure 4a). It is notable that the CLO peaks are still visible due to the distribution of the oil at the surface of the ECLO microcapsule, which is in agreement with the CLSM data (Figure 2a).

The FTIR spectra of the core–shell microcapsules (H-ECLO) did not exhibit main peaks of CLO and displayed similar spectra to the Hi–Cap, which further confirms the encapsulation of the fish oil within the core of the microcapsules (Figure 4c). 

### 3.5. Raman Analysis

**Figure 5:**
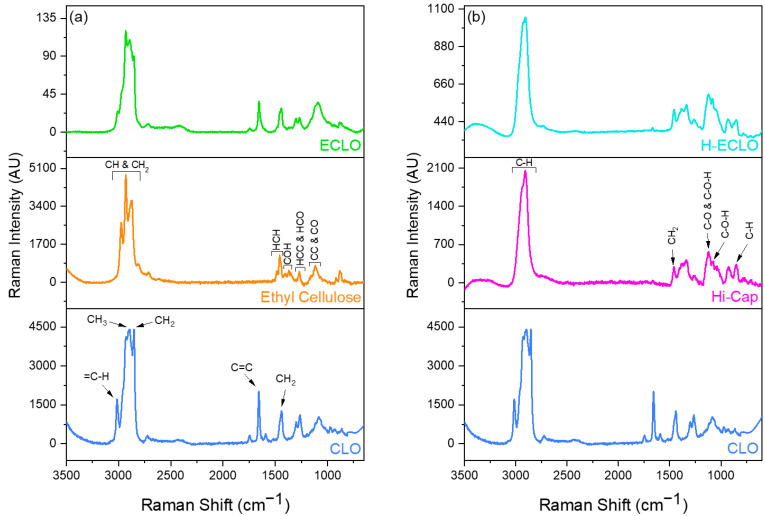
Raman spectra of (**a**) non–encapsulated cod liver oil (CLO), ethyl cellulose powder, and monoaxial electrosprayed (ECLO) microcapsules; (**b**) Raman spectra of CLO, Hi–Cap powder and coaxial electrosprayed (H–ECLO) microcapsules.

The Raman spectrum of the CLO is presented in Figure 5. The peak that appeared at 3014 cm^−1^ indicated =C–H groups’ stretching, which is characteristic of unsaturated fatty acids [60]. In the region of ~3000–2800 cm^−1^, C–H stretching can be observed; the band at ~2900 cm^−1^ is assigned to the CH_3_ groups and at ~2850 cm^−1^ to the CH_2_ groups [61]. The peak at 1656 cm^−1^ is related to the stretching vibration of C=C bands, and the peak at ~1440 cm^−1^ represents the CH_2_ group characteristics of the unsaturated lipids [61].

The Raman spectra of the ethyl cellulose (Figure 5a) exhibited several intense bands in the region ~3000–2800 cm^−1^, attributed to CH and CH_2_ stretching vibrations [62]. The region between ~1500 to 1430 cm^−1^ is related to HCH bending, the bands observed from 1430 to 1350 cm^−1^ are assigned to COH bending, and from 1350 to 1270 cm^−1^ to HCC and HCO bending. In the region between 1180 to 950 cm^−1^, the observed bands are related to CC and CO stretching motions [62]. Furthermore, ethyl cellulose displays peaks at ~1080 cm^−1^ which represents the gauche stretching of C–C bonds [61]. A peak appearing at a wavelength of ~920 cm^−1^ is associated with CH_2_ vibrations [61]. 

The Raman spectrum of ECLO (Figure 5a) shows the main peaks from the oil, confirming oil distribution at the surface of the microcapsules. Particularly, the peak at 1656 cm^−1^ is attributed to the stretching vibration of C=C bands related to the fish oil, which can be detected in the spectrum of the monoaxial electrosprayed microcapsules.

The Raman spectrum of the Hi–Cap (Figure 5b) shows main peaks in the region between 3000–2800 cm^−1^, which are assigned to C–H stretching [63]. The peak at 1460 cm^−1^ is assigned to CH_2_ bending, and the peaks at 1124 and 1082 cm^−1^ are assigned to C–O stretching and to C–O–H bending, respectively. The peak at 850 cm^−1^ is attributed to C–H deformation [63]. The Raman spectra of the H–ECLO microcapsules (Figure 5b) displayed only the main peaks observed for the Hi–Cap, while no bands of the CLO were detected on the surface of the microcapsules.

**Figure 6:**
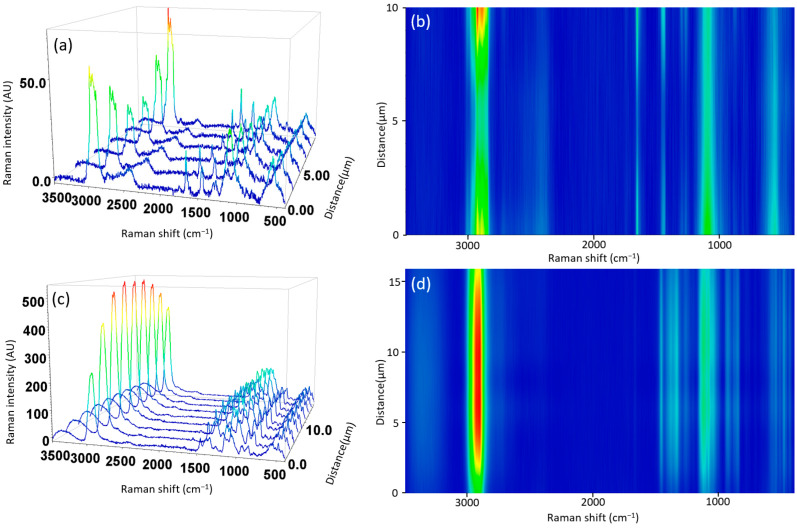
Line-scanning Raman mapping of 3D image spectra across the surface of the electrosprayed (**a**) monoaxial (ECLO) and (**c**) coaxial (H–ECLO) microcapsules and (**b**,**d**) corresponding contour map images.

Furthermore, the line-scanning Raman mapping spectra across the surface of the ECLO microcapsules (Figure 6a) and their contour plots (Figure 6b) showed oil distribution at the surface of the monoaxial electrosprayed microcapsules. In contrast, the line-scanning Raman mapping spectra and the contour plot of H–ECLO (Figure 6c,d) did not exhibit oil at the surface of the shell, which suggests proper encapsulation of CLO within the core of the microcapsule. 

### 3.6. Oxidative Stability of the Microcapsules

**Figure 7:**
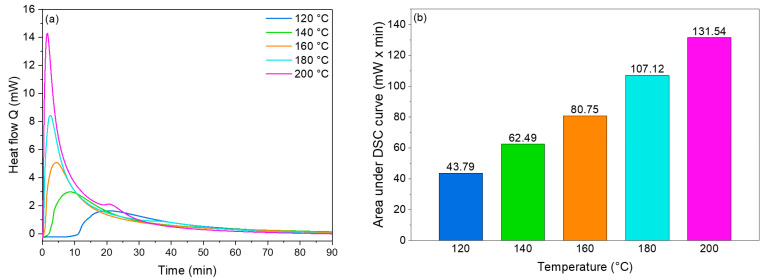
Oxidative stability of non-encapsulated CLO, as measured by DSC. (**a**) Exothermic DSC curves under different isothermal temperatures and (**b**) areas under DSC isothermal curves at different temperatures.

The oxidation of non-encapsulated CLO was evaluated by DSC isothermal analysis at different temperatures (120, 140, 160, 180, and 200 °C), as is shown in Figure 7a. After increasing the temperature, a higher exothermic peak and a decrease in the oxidative induction time (OIT) were observed [64]. The area under the isothermal curve was used to quantify and compare the oxidation of the non-encapsulated CLO (Figure 7b). After increasing the temperature from 120 °C to 140 °C, the area under the isothermal DSC curve increased by 1.4 times, and as the temperature was further increased to 200 °C, a three-fold increase in the area was observed.

**Figure 8:**
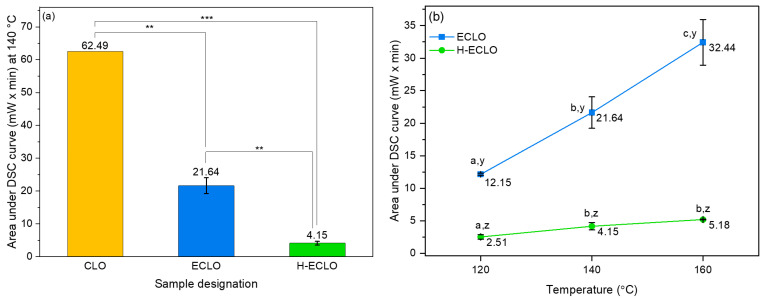
Oxidative stability of non-encapsulated and encapsulated CLO measured by DSC. (**a**) The area under the DSC isothermal curve calculated for the non-encapsulated CLO and for the monoaxial and coaxial electrosprayed microcapsules at 140 °C (two or three asterisks indicate significant differences between samples, with *p*-values of ≤0.01 and ≤0.001, respectively). (**b**) The area under the DSC isothermal curve at different temperatures for ECLO, and H-ECLO electrosprayed microcapsules. Values for the same samples with different lower caseletters (first alphabetic letters) are significantly different (*p*-value ≤ 0.05), and values within the same isothermal temperature for the different samples with different lower caseletters (last alphabetic letters) are significantly different (*p*-value ≤ 0.05).

The oxidative stability of the monoaxial and coaxial microcapsules was compared with that of the non-encapsulated CLO 140 °C (Figure 8a). The area under the DSC curve for the non–encapsulated CLO at an isothermal temperature of 140 °C was nearly 15 times higher (62.49 mW × min) in comparison to the area of the core–shell microcapsules (H–ECLO) (4.14 mW × min). However, the area under the isothermal curve of the non-encapsulated CLO revealed only a 2.9-fold decrease (21.64 mW × min) in comparison with the area of the microcapsules resulting from the monoaxial electrospraying (ECLO) (Figure 8a). Furthermore, the oxidative stability of the monoaxial and coaxial electrosprayed microcapsules at a temperature range of 120 °C to 160 °C is shown in Figure 8b. Significant differences were observed in the area under the isothermal curve of the ECLO and H–ECLO microcapsules. Particularly, the microcapsules produced from the monoaxial electrospray at the isothermal temperature of 160 °C showed a 6.27 times higher area in comparison to the microcapsules resulting from coaxial electrospraying (Figure 8b). Thus, the core–shell structure enhanced the oxidative stability of the oil by preventing its presence at the surface of the microcapsules, while the Hi–Cap shell also reduced the impermeability to oxygen, resulting in enhanced oxidative stability. Additionally, the ethyl cellulose also contributed to enhancing oxidative stability, most probably due to the hydrophobic interactions with CLO for both monoaxial and coaxial microcapsules (in agreement with the FTIR results). Previous studies have also shown that cellulose is able to trap reactive oxygen species (ROS); for example, this was observed in active and barrier multilayer films based on electrospun poly(3-hydroxybutyrate-co-3-hydroxyvalerate) and cellulose nanocrystal interlayers [65], as well as in native cellulose film polymerization to scavenge oxidative radicals [66].

Furthermore, the above data agree with the higher oxidative stability of the neat fish oil for microcapsules of maltodextrin with glucose syrup, which were prepared using coaxial electrospray processing, in comparison to monoaxial microcapsules. It was also suggested that the monoaxially produced microcapsules showed the highest oxidation due to their emulsification steps and a longer processing time [32]. Moreover, in our previous study, core–shell electrosprayed microcapsules of ethyl cellulose-Hi–Cap also showed an enhanced oxidative stability of the lipophilic vitamin A palmitate, mainly due to the absence of the bioactive compound on the surface of the microcapsules, as observed by DSC- isothermal analysis [33].

### 3.7. Numerical Simulation of the Electric Field

**Figure 9:**
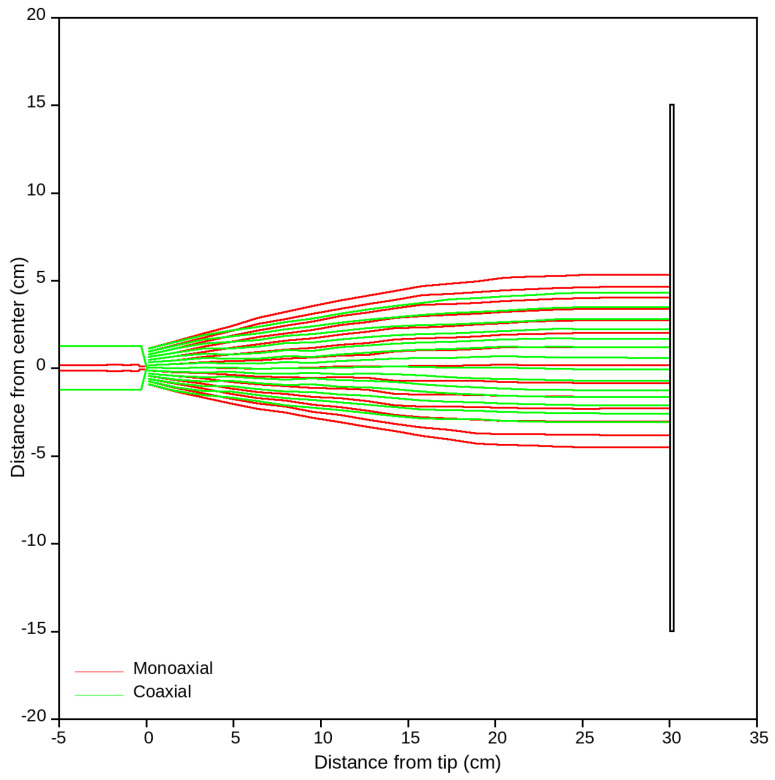
Electric field trajectories between the positively charged nozzle (40 kV) and collector (grounded) during the electrospraying process. The red line indicates monoaxial electrospraying and the green line indicates coaxial electrospraying.

The numerical simulation of the electric field strength and the electromagnetic potential were also compared for the monoaxial and coaxial electrospray processes based on the computational finite element method (FEM). The qualitative assessment of the electric field trajectories for the monoaxial or coaxial process between the needle/nozzle tip (40 kV) and the collector are illustrated in Figure 9. The monoaxial electrospray showed broader electric field paths compared to the coaxial electrospray, which showed narrower electric field paths. This narrow nature of the electric field in the coaxial electrospray process created a more concentrated and focused deposition of the core–shell microcapsules on the collector [48].

**Figure 10:**
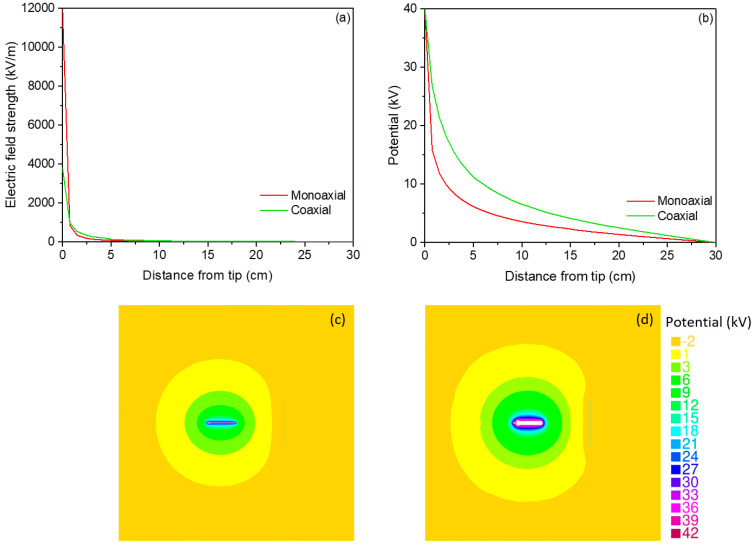
(**a**) Electric field strength and (**b**) the electric potential for the monoaxial (ECLO) and coaxial electrospray (H-ECLO) setups between the positively charged nozzle (40 kV) and the (grounded) collector during the electrospraying process. (**c**) The electric potential within the monoaxial and (**d**) in the coaxial electrospraying. The colors in (**c**,**d**) represent the potential in kV.

Quantitative evaluation of the electric field strength along the center line from the needle to the collector in the monoaxial electrospraying process indicated a sharper decline from 11,934 kV/m to 4 kV/m at the collector. However, for the coaxial electrospray, the electric field strength at the nozzle was much lower (3808 kV/m), ending at 23.8 kV/m at the collector (Figure 10a).

It has been reported that the shape and size of the needle affect the electric field. The smaller size of the needle leads to a stronger electric field close to needle [67,68]. This can probably explain the electric field differences observed in the monoaxial compared to the coaxial electrospray process. Likewise, the electric potential for the monoaxial electrospraying process showed a more abrupt reduction than the coaxial electrospraying process (Figure 10b). Moreover, the electric field strength distribution is also illustrated in Figure 10c,d for both the monoaxial and coaxial electrospraying process, respectively. 

Thus, the numerical simulation of the electric field gradient distribution between the nozzle tip and the collector revealed that a locally stronger electric field (close to the collector) was formed when the coaxial electrospraying process was utilized. This promoted the solvent’s evaporation and the deposition of the electrosprayed microcapsules on the collector as well as enhancing the oxidative stability, as observed for the core–shell microcapsules. 

## 4. Conclusions

The encapsulation of fish oil within ethyl cellulose core—OSA-modified starch shell coaxially electrosprayed microcapsules and their oxidation stability were evaluated. The H–ECLO core–shell matrix minimizes the amount of oil present at the microparticle surface, thus protecting it from oxidation, contrary to the ECLO capsules produced by monoaxial electrospraying using ethyl cellulose and fish oil. 

The areas under the isothermal DSC curves for non-encapsulated fish oil were nearly 2.9 and 15 times higher in comparison to the areas of the monoaxial and core–shell microcapsules, respectively, indicating the higher oxidative stability of CLO that is provided by the core–shell matrix. AFM studies also showed that the average surface adhesion and Young’s modulus of the H–ECLO core–shell microcapsules differed substantially from the ECLO microcapsules. Moreover, the FTIR analysis suggested that the hydrophobic interactions of fish oil and ethyl cellulose also enhanced the oxidative stability of the oil. 

The electric field strength distribution of both coaxial and monoaxial electrospray processes, was found to be substantially higher in the vicinity of the collector and lower in the proximity of the nozzle when the coaxial process was employed in comparison to the monoaxial process. This may also promote the solvent evaporation, the deposition of the core–shell electrosprayed microcapsules on the collector, and their oxidative stability. 

Overall, this study demonstrates that (hydrophobic) core—(amphiphilic) shell polysaccharide microcapsules could be utilized to modulate and enhance the oxidative stability of lipophilic bioactive compounds for nutritional and pharmaceutical applications. Furthermore, the addition of oxidation protection compounds in the core or in the shell of the microcapsules (such as hydrolysed proteins, fillers), could further enhance the oxidative stability of the encapsulated bioactive compounds and couldprovide additional functional, and oral delivery properties to the microcapsules.

## Data Availability

Data are contained within the article.
